# Effects of two exercise protocols on postural balance of elderly women: a randomized controlled trial

**DOI:** 10.1186/s12877-015-0059-3

**Published:** 2015-06-02

**Authors:** Laiana Sepúlveda de Andrade Mesquita, Fabiana Texeira de Carvalho, Lara Sepúlveda de Andrade Freire, Osmar Pinto Neto, Renato Amaro Zângaro

**Affiliations:** Universidade Camilo Castelo Branco –Unicastelo, São Paulo, SP Brazil; Associação Cidade da Ciência Tecnologia e Educação –CITÉ, São José dos Campos, SP Brazil; Universidade Estadual do Piauí- UESPI, Teresina, PI Brazil; Centro Universitário Uninovafapi, Teresina, PI Brazil; Rua Industrial Francisco Castro, 1622, Bairro Horto, Teresina, PI Brazil

**Keywords:** Movement techniques, Exercise therapy, Elderly, Physical activity, Rehabilitation

## Abstract

**Background:**

The aging process reduces both sensory capabilities and the capabilities of the motor systems responsible for postural control, resulting in a high number of falls among the elderly. Some therapeutic interventions can directly interrupt this process, including physical exercise. This study compares and examines the effects of two exercise protocols on the balance of elderly women.

**Methods:**

Elderly women who participated in a local church project (n = 63) were randomly divided into three groups: the proprioceptive neuromuscular facilitation group (PNFG), Pilates group (PG), and control group (CG). Of the 63 women, 58 completed the program. A training program involving 50-min sessions was performed in the PNFG and PG three times a week for 4 weeks. The elderly women in the CG received no intervention and continued with their daily activities. Stabilometric parameters, the Berg Balance Scale score, functional reach test, and timed up and go test (TUG test) were assessed before and 1 month after participation.

**Results:**

In the comparison among groups, the women in the PNFG showed a significant reduction in most of the stabilometric parameters evaluated and better Berg Balance Scale score, functional reach test result, and TUG test result than did women in the CG (*p* < 0.05). Women in the PG showed significantly better performance on the functional reach test and TUG test than did women in the CG (*p* < 0.05).

**Conclusions:**

Women in the PNFG showed significantly better static and dynamic balance than did women in the CG. Women in the PG also showed better dynamic balance than did women in the CG. However, no significant differences were observed in any of the balance variables assessed between the PNFG and PG.

**Trial registration:**

clinicaltrials.gov, number NCT02278731

## Background

Maintenance of the body’s balance is attributed to the postural control system, which involves motor, sensory and nervous system functions [1]. As age progresses, the degree of body sway increases even in simple postural forms, such as an upright posture, causing elderly individuals to sway more than young adults [2].

Changes associated with the aging process, especially those associated with balance, start around the age of 45 years and affect the sensory system (visual, vestibular, and somatosensory) and physical attributes (flexibility, strength, balance, and coordination) [3]. These changes also affect reaction time, causing the elderly to develop many balance-related issues. An improvement in balance control with regular exercise is a good strategy with which to successfully reduce the incidence of falls in this population [4, 5].

Several studies have verified the effect of proprioceptive exercise in increasing postural balance by increasing or decreasing body sway [4, 6]. Among several exercise protocols demonstrated to reduce the risk of falls in this population, proprioceptive neuromuscular facilitation (PNF) is particularly compelling because exercise programs aiming to improve balance in the elderly must involve coordination and proprioception activities in addition to strengthening [4, 7]. Some studies have indicated that PNF exercises are able to improve muscle strength and balance in the elderly [7, 8]. This approach to therapeutic exercises uses specific diagonal movement patterns, reproducing functional movements such as gait, to improve muscle strength and flexibility. It also uses sensory cues such as tactile, visual, and auditory stimuli to improve control and neuromuscular function [9, 10].

In addition to PNF exercises, the Pilates method, developed by Joseph Pilates, combines strength and flexibility training and has become increasingly popular, especially in rehabilitation programs [11]. One of the main objectives in Pilates is to strengthen the core muscles, also known as the “powerhouse”. This triggers stabilization of the back and pelvic muscles, maintains adequate spinal alignment against gravity, and provides support for limb movements [11]. Several studies have shown that when applied to the elderly, Pilates improves strength, mobility, coordination, and balance [12–14]. Alternatively, some contradictory results have been demonstrated. For example, Bird et al. [15] did not demonstrate significant differences in balance between a Pilates group and control group.

Considering the aforementioned information, we conclude that both PNF exercises and Pilates can improve the complex interaction among the systems responsible for postural balance in different ways. PNF exercises are performed with direct help from a physiotherapist who offers several stimuli to compensate for sensorial deficiencies in the elderly. In the Pilates method, however, the individual must continently control their movements [11] with indirect help from the physiotherapist, who only provides guidance on how to perform the movement.

Thus, Pilates and PNF are interesting choices for the elderly. Although performed differently, both methods stimulate the proprioceptive system, are easily reproduced and performed, and are low in cost. Few randomized controlled studies have investigated the influence of these types of exercises on the balance of the elderly population, and no studies to date have compared the efficacy of Pilates and PNF in improving static and dynamic balance in aged women.

Consequently, the aim of this study was to conduct a randomized controlled trial to investigate and comparethe effect of both exercise methods on the static and dynamic postural balance variables in elderly women, thus identifying alternatives to prevent falls and promoting functional independency.

## Methods

### Study participants

This study involved a convenience sample of older women belonging to a church project named “Pão dos Pobres”. The church was located near the physiotherapy department of a public hospital in Teresina, where the assessments and exercise sessions were conducted. Women who were sedentary as evaluated using the International Physical Activity Questionnaire and aged 60 to 80 years were included in the sample.

The exclusion criteria were any orthopedic, cardiovascular, vestibular, psychological, neurological, or other impairment that would not allow for the execution of all study tasks. A geriatrician who volunteered for the project examined all of the women before the evaluation and intervention.

The study was approved by the local ethics committee (Instituto Educacional Santo Agostinho, approval number 644.367), and all women provided written informed consent to participate. The study is registered on clinicaltrials.gov (number NCT02278731).

### Study design

In total, 150 women involved in the church project were invited to participate in the trial. However, only 90 were interested and therefore evaluated. After application of the inclusion and exclusion criteria, 63 women who could perform all proposed tasks were enrolled.

**Fig. 1:**
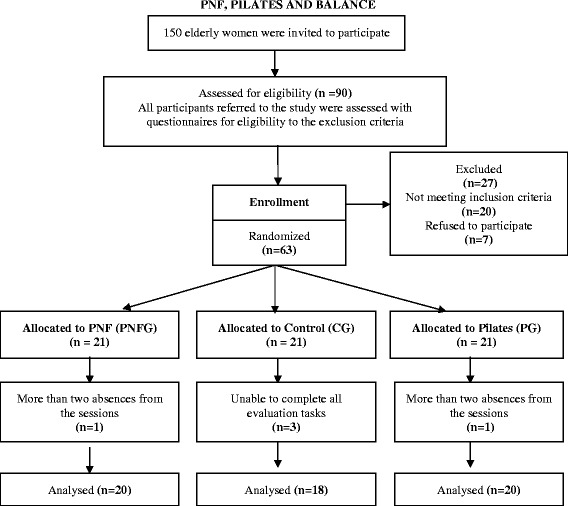
Study diagram and flow of participants

Each participant was randomly assigned by an independent researcher to either the Pilates group (PG), which involved 1 month of training in the Pilates method three times a week; the PNF group (PNFG), which involved 1 month of training in the PNF method three times a week; or the control group (CG), which involved no intervention (the participants continued their daily activities for 1 month). Three patients from the CG were unable to complete all evaluation tasks and one from the PG and one from the PNFG had more than two absences from the sessions and were thus excluded from the study analysis. The final sample distribution included 20 elderly women in the PNFG, 20 in the PG, and 18 in the CG. Fig. 1 shows the protocol distribution.

The calculations to establish the sample size were done using the software G*Power 3.1.9.2 with one-way ANOVA test in three groups. The significance level was set at α = 0.05 and power at 80 %. Partial eta squared (η_p_2 = 0.153) was used to measure the effect size, estimated at 0.425, according to the numbers set for the Timed Up and Go Test in a previous study [16]. The power analysis showed a minimum of 57 individuals (total sample size) were necessary to verify a significant intervention effect and 58 women completed the trial.

The delivery sessions lasted an average of 50 min. The Pilates and PNF protocols were delivered with progressive levels of difficulty to minimize the risk of pain or falls. The trial was performed from May to June 2014. Evaluations were performed 24 h before beginning the intervention and 24 h after its end. The protocols were delivered at the same previously determined time at a room temperature of 25 °C.

### Outcome measures

Data collection and data entry were conducted by research assistants who were blinded to the treatment conditions. Exercises were performed by two physiotherapy professionals. The PNFG provider was certified in PNF, and the PG provider was certified in Pilates.

#### Stabilometry

Data were collected using an electronic baropodometer (S-PLATE) comprising a force platform with 1600 sensors, active surface of 400 × 400 mm measuring 610 × 580 × 4 mm and 6.8 kg at the University Exercise Clinic in Teresina-PI. The women were oriented in the standing position on the platform; they were barefoot and stood with bipedal support and feet hip-width apart. Furthermore, they were instructed to keep their eyes open while staring at a specific point marked on the wall at eye level, arms relaxed on their sides, for 30 s. Women using corrective lenses wore them during the test. The platform was demarcated on the first attempt using adhesive tape, on which the women positioned their heels to assure the same basis throughout the tests, as suggested by Teixeira et al. [17]. The center-of-pressure (COP) sway kinetic variables analyzed with reference to balance were the total displacement oscillation (TDO), amplitude of displacement of the center of pressure in the anterior–posterior plane (ACPap), amplitude of displacement of the center of pressure in the mid-lateral plane (ACPml), displacement area (AREA), anterior–posterior average speed (AAS), mid-lateral average speed (MAS), and total average speed (TAS).

#### TUG test

The TUG test was performed to assess functional mobility regarding speed, agility, and dynamic balance of the elderly women according to Podsiadlo and Richardson [18]. This test assesses the level of mobility of the individual by measuring the time in seconds required to stand up from a chair (seat height of 45 cm) without the help of the arms, walk a distance of 3 m, turn around, and return to the chair. At the beginning of the test, the participants’ backs must touch the chair, and at the end of the test, the participants return to this position. Time was measured from the “go” command to return to the initial position. The test was performed once for familiarization prior to the timed trial. The women were instructed to perform the test as fast as possible while maintaining a comfortable speed to avoid accidents.

#### Functional reach test

The functional reach test was performed to analyze dynamic balance. The women were barefoot and instructed to position themselves orthostatically with their lateral body perpendicular to the wall but not touching the wall, feet parallel in a comfortable position, shoulders flexed at 90° (close to the wall), elbows extended, and hands closed. A measuring tape was fixed horizontally on the wall, parallel to the floor, and positioned at the height of the participant’s acromion. The initial measurement was taken at the position at which the third metacarpal met the measuring tape. The women were instructed to lean forward as much as possible without losing balance or taking a step, and the distance was measured as the displacement of the tape from the initial measurement at the third metacarpal to the final measure [19].

#### Berg balance test

The Berg Balance Test utilizes a scale of 14 common tasks involving static and dynamic balance, such as reaching, spinning, standing on one foot, and getting up. Each task is given 4 points on an ordinal scale, ranging from 0 (unable to perform the task) to 4 (performs the task independently). The scores of the 14 tasks are summed to yield a total score ranging from 0 to 56 points. Higher scores indicate better performance, while scores of ≤45 are predictive of falls [20].

### PNF intervention

The PNF diagonal patterns of movement were selected considering all the basic facilitation procedures, including resistance, manual pressure, traction, stretch and approximation reflexes, and visual and verbal stimulation. The patterns were consistent with the three specific principles of PNF: rhythmic initiation, sustentation and relaxation, and reversal of antagonists. Initially, the PNF hold-relax stretching technique was performed on the upper and lower limbs. The exercises were then started on the upper limbs in a bilaterally symmetrical agonist pattern: external flexion-abduction-rotation and internal extension-adduction-rotation; external flexion-adduction-rotation and internal extension-abduction-rotation. The procedure was then immediately performed on the lower limbs in a bilaterally symmetrical antagonist pattern: external flexion-adduction-rotation and internal extension-abduction-rotation (diagonal gait); internal flexion-abduction-rotation and internal extension-adduction-rotation, both diagonals involving variant flexion and extension of the knee [9]. With the woman in a lateral decubitus position, scapular and pelvic girdle exercises were performed in a combined symmetrical and reciprocal manner in the anterior-elevation-posterior-depression diagonal [9]. The researcher offered gradually increasing resistance throughout the range of motion, and the women thus gained strength. Delivery sessions were individualized. During the first week, 1 set of 10 repetitions for each diagonal was performed; in the second week, 2 sets of 10 repetitions were performed; and in the third and fourth weeks, 3 sets of 10 repetitions were performed.

### Pilates intervention

The Pilates protocol involved the performance of Pilates exercises on the ground. Stretches were performed on the upper body and lower limbs before the exercises. Exercises that involved the range of motion and strength of the upper limbs, trunk, and lower limbs were subsequently performed. All exercises were associated with breathing and contraction of the transverse abdominal muscles and were performed in different positions. The number of repetitions and level resistance were increased throughout the study using a Swiss ball, theraband, and magic circle. The exercise sessions were delivered in groups with up to three participants (Table 1).

**Table 1:** Pilates intervention

FIRST WEEK	REPETITIONS
Elevate the ball using upper limbs in dorsal position	5
Move the ball laterally to the right and left with the heels	5
Leap over the ball while seated	5
Belly dance	5
Bent-knee fall-out	5
Circle using legs in dorsal position	5
Bridge	5
Flex elbow against the wall	5
SECOND WEEK	
Elevate the ball using upper limbs in dorsal position	10
Move the ball laterally to the right and left with the heels	10
Leap over the ball while seated and lifting a foot	10
Walk on the ball while seated	10
Four-footed	10
Circle with legs in dorsal position	10
Bridge	10
Flex elbow against the wall while lifting a foot	5
THIRD WEEK	
Front/Back	5
Up/Down	5
Bicycle	8
Single leg circles, lying down with use of theraband	8
Sit on the ball, place theraband under feet, and perform shoulder flexion	10
Practice magic circle	10
Elevator	10
Lift the ball in the air	10
Four-footed on the ball while lifting hand/foot, alternating sides	10
Bridge while lifting a foot	10
Flex elbow against the wall while lifting a foot	10
Extend knee with magic circle	10
FOURTH WEEK	
Front/Back	10
Up/Down	10
Bicycle	10
Single leg circles, lying down with use of theraband	10
Sit on the ball, place theraband under feet, and perform shoulder flexion	10
Practice magic circle	10
Elevator	10
Lift the ball in the air	10
Four-footed on the ball, lifting hand/foot and alternating sides	10
Bridge while lifting a foot	10
Flex elbow against the wall while lifting a foot	10
Extend knee with magic circle	10

### Data analysis

A per protocol analysis was used to determine the most efficient of two exercise protocols (Pilates and PNF), lasting four weeks each, in a prearranged population of elderly women. Thus, the women with more than two absences from the sessions were excluded from the analysis. Data were processed and analyzed using PASW Statistics for Windows, Version 18.0. (SPSS Inc., Chicago, IL). Means and standard deviations are presented. First, the Shapiro-Wilk test was used to evaluate the normality of the variables and determine which tests would be used. For the variables following a normal distribution, the paired *t* test was used to compare two means (within-group comparisons), and repeated-measures ANOVA was used to compare three means (between-group comparisons). The Wilcoxon test was used on some stabilometric variables that did not follow the normal distribution. The differences in functional test scores and stabilometric variables were calculated for each woman by subtracting the pretraining data from the post-training data in the PG, PNFG, and CG. Repeated-measures ANOVA followed by post-hoc Tukey’s test was used to determine differences in the means of each variable between the groups. A p-value of <0.05 was considered statistically significant.

## Results

Sixty three elderly women were eligible for inclusion and 58 participants completed the program: 20 in the PNFG (age = 68.5 ± 5.4 years), 20 in the PG (age = 67.3 ± 4.9 years) and 18 in the CG (age = 71.5 ± 6.2 years). No differences in age, weight, height and body mass index were found between the three groups (Table 2).

**Table 2:** Homogeneity of women among the groups (n = 58)

	Control (n = 18)	PNF (n = 20)	Pilates (n = 20)	p*
Age (years)	71.5 ± 6.2	68.5 ± 5.4	67.3 ± 4.9	0.065
Weight (kg)	55.7 ± 5.7	58.4 ± 9.3	61.0 ± 9.3	0.164
Height (cm)	148.3 ± 7.0	152.7 ± 5.5	153.2 ± 5.9	0.335
Body mass index (kg/m^2^)	25.4 ± 2.9	25.1 ± 5.6	26.0 ± 3.5	0.747

Data are presented as mean ± standard deviation

*ANOVA

A total of 12 sessions were scheduled for each participant from the Pilates and PNF groups. And all the women included in the analysis attended all the sessions.

### Stabilometric data

The between-group comparisons showed that the PNF group had greater reductions in four of the seven sway measures than the control group (Table 3). No significant differences were found between the Pilates and control groups in any of the sway measures. In the within-group comparisons, there were no significant differences in the COP variables between the Pilates and control groups. However, the PNF group had greater reductions in five of the seven sway measures than the control group (Table 4).

**Table 3:** Comparative data of stabilometric and functional capacity (between-group comparison)

	Control	PNF	Pilates	p
	(Pos-Pre)	(Pos-Pre)	(Pos-Pre)	
TODO (mm)	148.8 ± 396.3	−165.9 ± 259.2*	−34.7 ± 230.9	0.008
ACPap (mm)	2.6 ± 5.5	−0.9 ± 5.2	0.4 ± 4.0	0.099
ACPml (mm)	0.6 ± 6.0	−4.2 ± 7.2	−2.4 ± 5.4	0.069
AREA (mm^2^)	40.0 ± 101.0	−37.2 ± 51.6*	−11.7 ± 38.6	0.003
AAS (mm/s)	4.4 ± 10.2	−1.6 ± 9.1	0.6 ± 6.7	0.115
MAS (mm/s)	1.7 ± 9.2	−6.1 ± 5.6*	−2.1 ± 4.9	0.003
TAS (mm/s)	5.0 ± 13.2	−5.5 ± 8.6*	−1.2 ± 7.7	0.008
BERG	0.5 ± 2.7	4.1 ± 4.5*	2.0 ± 1.9	0.005
TAF (cm)	0.3 ± 7.9	9.2 ± 4.8*	8.8 ± 5.0*	<0.001
TUG (sec)	0.1 ± 3.7	−2.6 ± 1.3*	−3.6 ± 2.3*	<0.001

Data arepresented as mean ± standard deviation of the pre- to post-test differences calculated as post minus pre so that positive means indicate an increase over time

*Significant difference compared with control group by Tukey’s post-hoc test after ANOVA, *p* < 0.05

TDO: total displacement oscillation, ACPap: amplitude of displacement of the center of pressure in the anterior–posterior plane, ACPml: amplitude of displacement of the center of pressure in the mid-lateral plane, AREA: displacement area, AAS: anterior–posterior average speed, MAS: mid-lateral average speed, TAS: total average speed

BERG: Berg Balance Scale score, TAF: functional reach test, TUG: timed up and go test

**Table 4:** Stabilometric results of elderly women before and after (within-group comparison) Pilates and PNF training or usual activity

	Pre	Post	P
Control			
TDO	701.5 ± 259.8	853.9 ± 432.7	0.129^t^
ACPap	11.1 ± 4.4	13.7 ± 6.6	0.063^t^
ACPml	8.6 ± 3.8	9.2 ± 4.8	0.660^t^
AREA	62.6 ± 36.3	102.6 ± 100.6	0.660^t^
AAS	17.2 ± 7.0	21.5 ± 12.1	0.086^t^
MAS	12.9 ± 5.7	14.6 ± 8.0	0.446^t^
TAS	23.5 ± 8.7	28.5 ± 14.4	0.129^t^
PNF			
TDO	820.6 ± 367.0	654.6 ± 350.6	0.019^w^
ACPap	11.7 ± 4.7	10.7 ± 4.1	0.601^t^
ACPml	11.5 ± 8.2	7.3 ± 5.4	0.001^w^
AREA	97.5 ± 57.5	60.3 ± 98.3	0.003^w^
AAS	18.8 ± 8.6	17.2 ± 7.6	0.654^w^
MAS	16.2 ± 9.2	10.1 ± 8.0	<0.001^w^
TAS	27.4 ± 12.2	16.4 ± 11.7	0.019^w^
Pilates			
TODO	629.5 ± 213.6	594.6 ± 235.4	0.510^t^
ACPap	8.9 ± 3.3	9.3 ± 2.5	0.601^w^
ACPml	9.4 ± 5.4	7.0 ± 2.7	0.117^w^
AREA	55.8 ± 37.6	44.1 ± 32.1	0.191^t^
AAS	14.4 ± 4.6	15.0 ± 6.5	0.701^t^
MAS	12.3 ± 6.2	10.2 ± 4.8	0.072^t^
TAS	21.0 ± 7.1	19.8 ± 7.8	0.510^t^

Data are presented as mean ± standard deviation

^t^Student’s *t* test, ^w^Wilcoxon tes

TDO: total displacement oscillation, ACPap: amplitude of displacement of the center of pressure in the anterior–posterior plane, ACPml: amplitude of displacement of the center of pressure in the mid-lateral plane, AREA: displacement area, AAS: anterior–posterior average speed, MAS: mid-lateral average speed, TAS: total average speed

### Functional tests

In the between-group comparisons, women in the PNFG and PG exhibited improved performance in the TUG test and functional reach test compared with women in the CG. The Berg Balance Scale scores improved in the PNFG when compared with the CG (*p* = 0.005). No significant differences in any functional test results were observed between the PG and PNFG (*p* > 0.05) (Table 3).

In a within-group comparison, women in both the PNFG and PG showed significant improvements in the functional reach test, TUG test, and Berg Balance Scale scores. Women in the CG did not show significant differences in the evaluated parameters (Table 5).

**Table 5:** Functional tests of elderly women before and after (within-group comparison) Pilates and PNF training or usual activity (control)

	Pre	Post	p*
Control			
BERG	50.5 ± 3.4	51.0 ± 3.5	0.462
TAF (cm)	19.7 ± 7.6	20.0 ± 6.5	0.878
TUG (sec)	13.8 ± 3.3	13.9 ± 4.3	0.915
PNF			
BERG	51.7 ± 4.8	55.8 ± 0.4	0.001
TAF (cm)	21.7 ± 7.3	30.9 ± 5.7	<0.001
TUG (sec)	10.7 ± 1.9	8.1 ± 1.9	<0.001
Pilates			
BERG	54.0 ± 1.9	56.0 ± 0.1	<0.001
TAF (cm)	24.6 ± 7.0	33.4 ± 5.9	<0.001
TUG (sec)	11.3 ± 2.5	7.7 ± 1.5	<0.001

Data are presented as mean ± standard deviation

*Significant difference by Student’s *t* test

BERG: Berg Balance Scale score, TAF: functional reach test, TUG: timed up and go test

## Discussion

This study represents the first controlled evaluation comparing two physiotherapy treatments in the balance of elderly women: Pilates and PNF. The results suggest that in 12 sessions, both methods improved parameters related to the postural balance of elderly women; however, there were no differences between the PNFG and PG. There was a greater reduction in COP sway, verified through stabilometric parameters, in the PNFG than in the CG. The PNFG also showed significant improvements in all functional tests performed. Although women in the PG did not show a significant reduction in COPsway, they showed a significant improvement in the functional reach test and TUG test performance.

Several stabilometric parameters derived from the COP have been used to quantify changes in balance. The selection of these parameters has been controversial in the literature; there are conflicting opinions about which parameters are more sensitive to fluctuations in the COP. Understanding these variables is essential for analysis of balance [21].

Fujimoto et al. [22] found that the area of oscillation may indirectly reflect the function of the central and peripheral vestibular systems due to reduced visual and somatosensory input. In another study, the authors examined the reliability of COP measures, and the average speed was the most frequently used and most reliable parameter with which to evaluate the body sway [23]. This parameter was also more sensitive in discriminating the effects of manipulative treatments and challenges of balancing tasks for both young adults and healthy elderly participants [24]. The amplitude of COP displacement in the anterior–posterior and mid-lateral planes was also frequently used to verify the balance performance after proprioceptive training [15, 25]. Ruhe et al. [23] noted that although the average speed has good reliability across different studies, no single measure of COP appeared to be significantly more reliable than the others.

Although no significant differences were shown between the PNFG and PG, use of the PNF protocol in elderly women proved to have positive effects; several stabilometric variables demonstrated increased stability and static balance in this group after 12 weeks of exercise. Some studies have in dicated that the muscles respond to application of the PNF hold-relax stretching technique, suggesting that in addition to promoting gains in flexibility and increases in the range of motion, PNF also increases the muscle strength of the knee flexor and extensor muscles [26]. Furthermore, during the execution of the PNF movements, the muscles are briefly stretched before contraction, stimulating neuromuscular endings that produce greater strength. When exercises are performed using a diagonal pattern, which is parallel to the muscle topography, more physiological and functional movements are reproduced, such as gait [27]. The lower-limb PNF exercises were performed using the bilaterally symmetrical antagonist pattern, which not only strengthens the muscles involved in gait, but also allows for irradiation of the most powerful member to facilitate the movements of weaker muscles [9].

Among the reductions observed in the stabilometric parameters in the PNFG, the COP variables in the mid-lateral plane (ACPml and MAS) achieved statistical significance in the PNFG, while a significant reduction was not observed in any COP parameter in the anterior–posterior plane. This may have been a consequence of the use of a diagonal pattern, which allows the exercises to always be performed in combination with hip abduction and adduction and ankle inversion and eversion, stimulating the afferent somatosensory and lateral and middle muscular groups of the lower limbs and conferring greater stability to the mid-lateral plane.

Although no studies in the literature have utilized stabilometric variables to evaluate balance in elderly women after PNF exercises, Pereira et al. [7] evaluated the risk of falling among elderly people after PNF exercises were performed three times a week for 10 weeks. They verified significant improvements in the Berg Balance Scale scores and greater strength in the extensor compartment of the knee. Cilento et al. [8] also conducted a prospective study to comparatively evaluate the effectiveness of three training protocols in elderly women randomly selected and divided into four groups: a traditional muscle strengthening group, functional circuit training group, PNF exercise group, and control group. The elderly women participated in 30-min sessions twice a week for 10 weeks. At the end of the 10 weeks, they were evaluated using the TUG test and functional reach test. Statistically significant improvement was observed in all groups compared with the control group. In the present study, women in the PNFG exhibited significant improvements in the Berg Balance Scale scores, functional reach test results, and TUG test results.

Some studies have suggested that a better balance between agonist and antagonist muscle activation is achieved after PNF exercises, reducing coactivation [9, 27]. Research shows that increased coactivation around the knee and ankle is associated with greater strength and lower torque in elderly people [7, 27, 28]. Coactivation, defined as the simultaneous contraction of agonist and antagonist muscles around a joint, is responsible for reductions in walking speed and increases in the risk of falls in the elderly [28, 29]. Therefore, we can explain the improvement in static balance through the reductions in the stabilometric parameters and the improvement in dynamic balance through the improvements in the Berg Balance Scale scores, TUG test performance, and functional reach test performance in the elderly women after the PNF exercise protocol in the present study.

Another common exercise currently recommended and used in this study is the Pilates method, which emphasizes principles such as strengthening of the abdominal and paraspinal muscles in parallel with good posture, breathing, and body alignment [30]. The Pilates method effectively improves postural stability in elderly individuals because it emphasizes trunk control [14]. A strong and stable trunk can contribute to more efficient use of the upper and lower limbs and thus better balance and functional performance in older adults [4].

Although women in the PG did not show significant improvements in the COP parameters, they exhibited improvements in the performance of functional tests for dynamic balance. The exercise protocol for this method challenges balance. According to Sherrington et al. [5, 31], exercises performed for fall prevention should provide a moderate or high challenge to balance. Moreover, this technique helps to strengthen the abdominal muscles [32], which are important for a good posture. The strengthened abdominal muscles reduce anterior pelvic inclination, common in this age group, thus improving gait [33].

In a randomized study, Bird et al. [15] evaluated the static and dynamic conditions of elderly residents in a community sample through evaluation of the ACPml and performance of the TUG test, comparing the Pilates method with a control method. No significant improvement was observed in any measured COP variables between the two groups. However, within-group comparison revealed an increase in the TUG test performance (*p* < 0.001) in the Pilates group. Likewise, in the present study, there was a significant improvement in dynamic balance.

Kaesler et al. [12] examined the efficacy of a Pilates program specifically designed to improve balance. The sample comprised eight elderly adults (four female and four male). The sessions were performed twice a week for 8 weeks. As in the present study, no significant improvement was found in COP on a firm surface. However, the researchers verified better performance on the TUG test after the proposed treatment. Newell et al. [13] executed Pilates training with nine elderly adults (eight female and one male) once a week for 8 weeks. There was no significant difference in the COP body sway pre- and post-treatment (*p* > 0.05), but there was an increase in the walking speed, cycle, and stride length. Mokhtari et al. [14] found a significant improvement in the TUG test and functional reach test performance among elderly adults who participated in Pilates three times a week for 12 weeks when compared with a control group.

Postural balance results from complex interactions among the sensory, nervous, and musculoskeletal systems and requires constant adaptations of muscular activity, the articular position, and other sensorial information to maintain the center of body mass on the support base [34]. Pilates exercises are based on movement control, which can lead to changes in the nervous system through alterations in synaptic connections and cortical remapping [35].

The results of the present study demonstrated improvements compared with the CG, particularly in dynamic balance in the PG and static and dynamic balance in the PNFG. This may have occurred because the PNF exercises were performed with manual resistance provided by a therapist, eliciting maximum motor recruitment through the stretch reflex, manual contact, verbal stimulation, force irradiation, and visual-auditory biofeedback, all of which improve sensory compensation in the elderly as suggested by previous clinical studies [9, 10]. Pilates is an exercise method that requires contraction and coordination of multiple muscle groups to achieve better motor recruitment, as well as synchronization of body movements and breathing [11]. Thus, the elderly may need a longer duration and greater number of sessions to learn and maximally benefit from the Pilates protocol.

Furthermore, as this is a per protocol analysis reflecting the benefits of these two exercise methods in a predetermined population, the effect of the interventions can not be applied to real life scenarios.

## Conclusions

Women in the Proprioceptive Neuromuscular FacilitationGroup demonstrated improvement in postural static stability as shown by evaluation of various stabilometric parameters. We also found a significant increase in dynamic balance as verified by the functional test performance in the Pilates and Proprioceptive Neuromuscular Facilitation groups. After only 4 weeks of these exercises, an improvement in balance could be observed, although without differences between the Pilates and Proprioceptive Neuromuscular Facilitation groups. Therefore, we recommend that further studies include larger samples of elderly women and greater numbers of sessions. This will help to elucidate the optimal alternatives that can be applied to increase balance, allowing PNF and Pilates exercises to be used not only for rehabilitation, but also as a preventive method.

## Abbreviations

PNFG: Proprioceptive neuromuscular facilitation group
PG: Pilates group
CG: Control group
TDO: Total displacement oscillation
ACPap: Amplitude of displacement of the center of pressure in the anterior–posterior plane
ACPml: Amplitude of displacement of the center of pressure in the mid-lateral plane
AREA: Displacement area
AAS: Anterior–posterior average speed
MAS: Mid-lateral average speed
TAS: Total average speed
TUG test: Timed up and go test
